# Social Representations of Urinary Incontinence in Caregivers and General Population: A Focus Group Study

**DOI:** 10.3390/ijerph191912251

**Published:** 2022-09-27

**Authors:** Lea Peroni, Didier Armaingaud, Tassadit Yakoubi, Monique Rothan-Tondeur

**Affiliations:** 1 University Paris 13, Sorbonne Paris Cite, Nursing Sciences Research Chair, Laboratory Educations and Health Promotion (LEPS), (UR 3412), UFR SMBH, F-93017, 1 rue de Chablis, 93000 Bobigny, France; 2 Korian Foundation for the Ageing Well, 21-25 rue Balzac, 75008 Paris, France; 3 AP HP, Nursing Sciences Research Chair, 55 Boulevard Diderot, 75012 Paris, France

**Keywords:** social representations, urinary incontinence, focus groups, nursing homes

## Abstract

Urinary incontinence (UI) is a major public health problem. Although trivialized, it affects nearly 9% of the world’s population and its prevalence increases with age. It affects many people living in nursing homes. In the literature, there is a lot of information on its symptoms, risk factors and therapeutic approaches, but its social representations are rarely studied. The objective of this qualitative focus-group study is to understand the social representations of urinary incontinence of caregivers of institutionalized elderly people, but also of the general population. Seven focus groups were organized with 41 participants. The data collected were analyzed both manually and using Atlas.Ti software. For caregivers and the general population, urinary incontinence remains a misunderstood and disturbing subject: judged as too intimate, embarrassing, and shameful, it is even considered uninteresting by those who are not affected, with some going so far as to make fun of those affected. It is also represented as a real difficulty for relationships: it can be a source of conflict, but also of questioning by the role of caregiver. However, it is also represented as a means of increasing the empowerment of the residents concerned, thanks to the choice of their means of protection. This study has enabled us to gain a deeper understanding of the social representations of caregivers and the general population on urinary incontinence in the elderly, but also to highlight the various preventive and educational actions that could be taken to improve the management of this health problem. It is part of a larger research program that aims at understanding the representations of urinary incontinence of caregivers, the general population, but also of course, of residents in nursing homes and their carers.

## 1. Introduction

Urinary incontinence is defined by the International Continence Society as “an involuntary and uncontrollable flow of urine through the urethra”. Several types of urinary incontinence have been defined according to its mechanism of occurrence: stress urinary incontinence, bladder overactivity incontinence, and mixed urinary incontinence. The literature identifies numerous risk factors: age, number of pregnancies, obesity, diabetes, certain neurological diseases, but also prostate hypertrophy, the use of certain medications, alterations in bladder and bowel functions, or physical dependence and mental impairment [1]. UI is a major public health problem affecting at least 7.84% of women and 3.08% of men, i.e., almost 9% of the world population [2]. Among them, people over 65 years old are mostly represented. Indeed, the prevalence of UI increases with age and the anatomical changes linked to the ageing of the individual, as well as comorbidities, predispose the elderly to this type of health problem [3]. In nursing homes, the figures vary from 30 to 80% depending on the institution. However, urinary incontinence is also higher in women than in men, although this difference tends to diminish with advancing age [4]. This health problem also has many consequences, both for the person affected and for their caregivers and relatives. The physical consequences are important, but the psychological consequences are also very strong: anxiety, depression, social isolation, to name but a few [5]. Their caregivers find themselves helpless in the face of this problem, as support requires time [6]. It is sometimes perceived as tiring and can affect their own quality of life [7]. In nursing homes, many elderly people already feel socially isolated. Indeed, several studies describe feelings of loneliness, abandonment, or exclusion [8,9,10]. Their social interactions are limited, and urinary incontinence seems to increase these painful feelings. In the literature, urinary incontinence is well defined [11]. A lot of information is given on its diagnosis, risk factors, consequences, or therapeutic alternatives. However, there is very little information about its representations. However, a Swiss study has shown that a better understanding of the social representations of this phenomenon could improve the management of affected patients [12]. Indeed, studying social representations of urinary incontinence can allow us to understand the beliefs, opinions or prejudices that exist on the subject, and thus to adapt the actions to be taken to improve knowledge on this problem. It is through this learning that the care of institutionalized elderly people concerned by UI can be improved.

Social representations are defined as “a form of knowledge, socially elaborated and shared, having a practical aim and contributing to the construction of a reality common to a social group” [13]. This definition makes it possible to understand that an object, a situation or even a phenomenon will be interpreted by each individual according to his or her own vision of the world; the latter is dependent on personal history, the social context in which a person evolves or the values in which the person believes. Social representations are dictated by our common sense [14], and not by precise knowledge. They are necessary for each individual, because they allow a person to understand an environment in order to act in the best possible way. To study the existing social representations on urinary incontinence of institutionalized elderly people, this study is based on Abric’s conceptual model, and more specifically on his theory of the central core and the silent zone [15]. On the one hand, according to this author, a social representation is “an organized set of information, opinions, attitudes and beliefs about a given object” [15]. To describe what one of these representations is about is to define the way in which an object is thought of by a community [16]. The silent zone, on the other hand, is described as a socially inexpressible zone [17]. Exploring the latter makes it possible to obtain, on a subject as sensitive as urinary incontinence, elements of understanding. In fact, this information is not spontaneously mentioned by the people interviewed, as it is considered too embarrassing. Some speech seems unacceptable to other individuals. Several authors therefore recommend that, in order to gain access to this zone, people should be asked to answer questions associated with others [18]. The aim of this study was therefore to understand the social representations of urinary incontinence of caregivers of the institutionalized elderly, but also of people in the general population.

## 2. Materials and Methods

### 2.1. Design

The aim was to carry out a qualitative study with a descriptive and comprehensive aim, using a phenomenological approach [19] and focus groups [20]. An interview guide was developed in order to obtain information on urinary incontinence, but also on the means of protection.

### 2.2. Population

**Figure 1:**
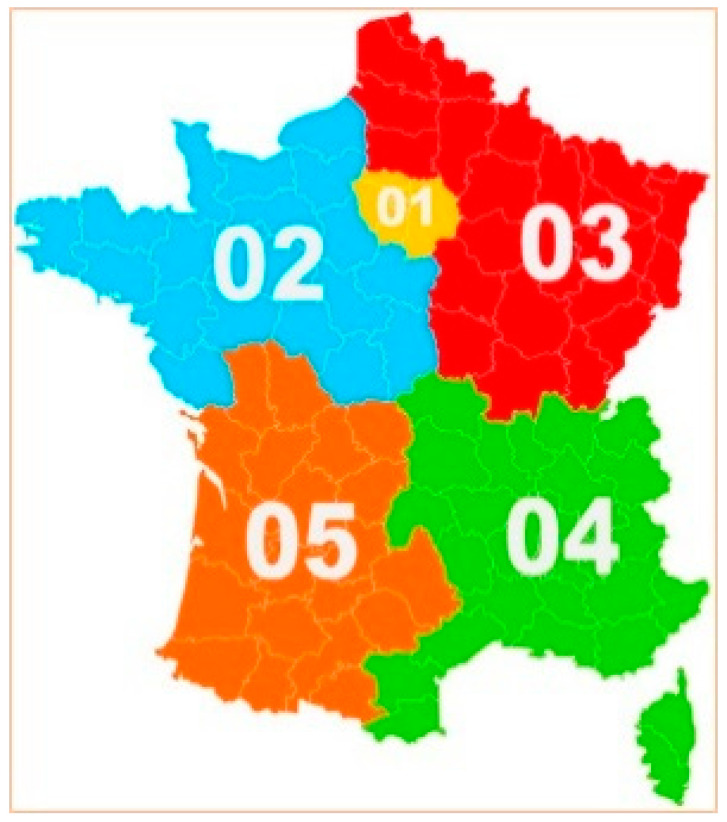
Representation of the five French regions delimited for the qualitative study by focus groups.

In order to obtain the representations of the French population on this subject, five large geographical regions were delimited see Figure 1: Ile de France (1), North-West (2), North-East (3), South-East (4) and South-West (5).

The next step was to conduct a focus group in each of these regions. After a call for participation from public and private nursing homes in the five regions, a list of volunteers was drawn up, and seven of them were then chosen at random. This number of nursing homes was chosen in order to represent the French population as closely as possible. The researchers then asked the nurse coordinators of these facilities to distribute information notes and to recruit a minimum of three caregivers and three people from the general population to participate in focus groups. A caregiver was considered to be any person in the resident’s entourage who voluntarily provided physical or psychological assistance. A person from the general population was considered to be any individual who had no connection with the residents. He or she was not to be a caregiver or a carer. To be included in the study, participants had to be at least 18 years old and able to understand, answer questions and exchange with other members of a group.

### 2.3. Sampling

Within the seven randomly selected facilities, caregivers and people from the general population were approached through the nurse coordinators. The sample of caregivers and people from the general population was therefore non-random and incidental, and seven focus groups were conducted, i.e., one focus group per facility.

### 2.4. Implementation and Analysis

The seven focus groups were conducted between July and October 2021. As recommended in the literature, data collection was carried out by three people [21]: a moderator, who led the focus group and followed a previously prepared interview guide, an observer, whose task was to take notes on the expressions and nonverbal attitudes of each participant, and a secretary, whose role was to take notes of all the exchanges between the participants. Each focus group began with a friendly welcome of the participants by the whole team. The participants were received in a quiet, spacious environment that was conducive to discussion [22]. Once settled, an information note was distributed to them. A sign-in sheet was then filled out by the participants in order to obtain socio-demographic data (gender, age, region, marital status and socio-professional category) as well as information on their relationship with the subject (caregiver, general population). Afterwards, the facilitator presented the reason for the meeting in a concise manner and asked the participants to give their oral consent for the audio recording of the exchanges. Each discussion group then had the same structure. The questions in the interview guide provided information about the components of the participants’ representation. In the first stage, the questions focused on their knowledge and opinions about urinary incontinence, and in the second stage, they were asked to interact about means of protection. Substitution techniques [15] were used to identify the “central core” and the “silent zone” of the representations.

The focus groups simultaneously generated data for three units of analysis: individual, group, and participant interaction. This allowed for the visualization of the consensus march and the notion of cohesion [23]. Before beginning any analysis, the entire exchange between the participants had to be transcribed word for word, without any modification, interpretation or abbreviation of the text [24]. A first researcher therefore carried out this transcription. The first analysis of the data was manual. It aimed at highlighting the characteristics of the interviewees, the codes, the themes and, in total, the phenomena of importance. It was therefore descriptive. The next step was to carry out an axial coding of the verbatim [25]. Each of the verbatim was classified into a category that respected the idea that was brought up. These categories were then grouped into themes. This step helped us to determine the extent to which the analyzed data contributed to data saturation, and increased descriptive, interpretive, and theoretical validity, which in turn increased understanding of the social representations of older adults’ UI. In a second phase, an analysis was carried out using the Atlas Ti version 9 qualitative data analysis software (Scientific Software Development GmbH, Berlin, Germany). This phase was more comprehensive, it was the interpretative analysis of the data. It allowed for the analysis of the content of the verbatim, the structure and the polarity of the terms used. It also allowed the calculation of polarity indices as well as the frequency of appearance of words and groups of words, a comparison of the data in the literature could be carried out and could lead to the formulation of hypotheses. Finally, a triangulation of the data was performed [26]. Thus, the information obtained was compared by four researchers, which made it possible to bring reliability to the analyzed data.

### 2.5. Ethical Considerations

This study was carried out in accordance with the good research practices of the Nursing Sciences Research Chair and in compliance with current French research regulations. In addition, the protocol for this study was reviewed by the Ethics and Research Committee and received its approval on 12 July 2021. It has the number IRB 00012021-43. It was also registered with the Research Registry, under the identification code researchregistry6965.

Prior to each focus group, an information letter was presented to each participant. Afterwards, their oral consent was collected and recorded. To ensure confidentiality and anonymity of the data collected, a code was assigned to each participant. Finally, only relevant and necessary data for the study were collected and processed.

## 3. Results

### 3.1. Characteristics of the Participants

A total of 41 people participated in the seven focus groups: 19 of them were caregivers of institutionalized elderly people, and 22 of them were people from the general population. In each of the focus groups, there were between five and seven participants. There was a real heterogeneity of sociodemographic profiles Table 1. The average age of the participants was 54.73 years, with ages ranging from 23 to 97 years. Regarding their socio-professional category, the participants were divided into categories 3, 4, 5 and 7, corresponding, respectively, to executives and intellectual professions of higher education, intermediate professions, employees, and retired people. However, two points seem important to raise: 90.24% of the participants (i.e., 37 out of 41) were women, and the 19 caregivers could be grouped into three distinct categories. Some were spouses of the institutionalized elderly people, some were children, and some were family members.

The main results of this focus group study showed that caregivers and people from the general population have many representations, both of urinary incontinence and of means of protection.

### 3.2. Social Representations of Urinary Incontinence

#### 3.2.1. A Poorly Understood Subject

For the participants in the 7 focus groups, UI is an unknown subject. Indeed, despite some limited knowledge about its definition, its causes, and the people affected, there are many false beliefs. Two of these were raised repeatedly during the discussion and may have a significant impact on the public’s representation of urinary incontinence. The first is that UI is systematically associated with dependence. In all the focus groups, the health problem was seen as a loss of independence. It was not envisaged that a person with urinary incontinence could be independent. For example, one participant said, “When you get up, it’s not incontinence!” (FG7/C18). The second misconception dealt with the fact that urinary incontinence would affect everyone. In fact, one of the participants very clearly said “everyone gets it!” (FG1/GP2). In addition, it is interesting to note that the participants expressed many questions and doubts, mainly about the physical and psychological causes of this health problem. Caregivers, for their part, had twice as many questions about urinary incontinence as people in the general population, but overall, many participants were taking notes during the exchanges.

#### 3.2.2. A Disruptive Part of Daily Life

UI was also represented as a disruptive element in the daily lives of those affected. In particular, participants identified many physical problems. For the caregivers, as for the general population, the major problems are dependence, cited in five focus groups out of seven, and odour problems, also cited in five focus groups out of seven, (“you can’t stand next to me anymore because there’s an odour problem, we can’t stand in a mini room like that for 2 h”-FG6/C14). They also denounced problems with tasks, discomfort, falls, cystitis and leaks, with participants going so far as to use the term “overflow”. Participants also spoke of the psychological consequences of urinary incontinence. Most of them mentioned the fear of going out (mentioned in five focus groups out of seven), the stress and anxiety linked to this health problem and the isolation. It would also seem that urinary incontinence causes many problems with others: some consider that urinary incontinence limits the time spent with others and leads to a real lack of socialization. For others, this desocialization is due to the rejection experienced by the incontinent person: “and then there is the rejection of the… well… I can imagine that some people reject the incontinent person” (FG5/C12). Still others mention the mockery that is directed at people with urinary incontinence. One caregiver, whose husband is urinary incontinent, talks with great sorrow about the fact that her friends no longer come to see her institutionalized husband: “Oh the mockery! Yes, the mockery! Because some people make fun. Why don’t our friends come to see my husband? Because he stinks sometimes! Well, you have to say what is, but that’s how it is” (FG6/C16). Finally, the subject of the alteration of the self-image is mentioned in all the focus groups, both by the caregivers and by the people from the general population: “it totally impacts their own image, because they don’t necessarily recognize themselves” (FG5/GP16).

#### 3.2.3. A Difficulty in Relationships

UI was also seen as a difficulty in relationships. Indeed, the role of family caregiver was often questioned during the discussions. As urinary incontinence is an intimate topic, participants expressed great difficulty in caring for their urinary-incontinent loved one, due to this very health problem. For example, one caregiver said, “I was no longer in my role…it’s normal for a parent to help his or her child, but for the child at this stage to come and help the parent, when he or she is sick, is okay, but that’s too intimate” (FG1/C1). The caregivers participating in the focus groups wondered if their role as family caregiver had reached a limit in the face of this UI. In addition, parent/child relationships were also often altered by this health problem and were described as potentially conflicting. In the participants’ speeches, intra-family conflicts between caregivers and their incontinent parent, but also between relatives of the same family concerning their incontinent parent: one caregiver specified that her mother’s behaviour irritated her (“Mom, she was holding… her buttocks. What does that look like? I would get a little angry because it annoyed me”-FG7/C17), while another caregiver said that their mother’s urinary incontinence created conflicts with her siblings.

#### 3.2.4. A Disturbing Subject

In almost all the focus groups (6/7), the participants raised the fact that UI is a taboo subject. The word itself was mentioned by 17 participants out of 41. UI was also said to be hidden because it was too intimate and private: “It’s too intimate to talk about very easily, openly, without embarrassment” (FG1/C1). Whether it is caregivers, people from the general population or incontinent people themselves, the subject remains a secret: “Even between women, or with one’s best friend, or with one’s husband, we won’t talk about it… it’s really something we don’t talk about” (FG3/GP11). In several focus groups, participants explained that it seems that the subject of UI is even more taboo for men than for women: “He never wanted to bother the care assistant. It was nonsense but… instead of calling, because…. She’s a man and he had a sense of humiliation” (FG7/C19). Others also find the topic more taboo for younger women than for older women: “Maybe it is more easily brought up, at least we hear about it more easily, concerning older people, than for much younger women” (FG7/GP22). One main reason for this taboo was raised during the focus groups is shame, with regard to the way society looks at this health problem. One participant expressed herself as follows: “When we went to the pharmacy, we always had to bring big packets! I was a bit ashamed to bring that” (FG7/C17). And the subject even seems sometimes difficult to discuss with health professionals: “My mom, she never went to the pharmacist. There’s no way the pharmacist would know I need sanitary napkins” (FG1/C2). However, it should be noted that in three of the focus groups, participants emphasized the fact that the subject of urinary incontinence was being discussed more and more, thanks in particular to the advertisements seen on television: “In relation to the advertisements, I was going to say that we are talking about it more and more” (FG5/GP15). In addition, it seems important to point out that participants believe that people in the general population imagine that it is a trivialized problem related to the elderly, that they are not concerned and that they sometimes come to make fun of them. Finally, the participants raised several differences: generational, in terms of attitudes and behaviors first of all, but also between men and women.

#### 3.2.5. A Transitional Stage

According to the participants, it would also seem that UI in institutionalized elderly people is considered a real transition in the lives of the people concerned. For the participants of three focus groups, this health problem constitutes “the beginning of old age” (FG2/C4), the “beginning of dependence” (FG4/GP12), “a complicated passage” (FG5/GP16), “before the turning point” (FG5/C12), but also the “beginning of a mourning process for the person they once were” (FG5/GP16). Adaptation and acceptance would therefore be necessary in the face of this new reorganized daily life, both for the urinary incontinent elderly person, but also for their caregiver. Finally, a parallel with the return to childhood was also drawn by the participants. A parallel was drawn between the elderly and children in four focus groups. This is linked to the use of protective clothing and to the fact that the person must be accompanied to the toilet. Some participants mentioned a feeling of regression when faced with this situation: “because it refers to childhood… regression, childhood…” (FG4/GP12). Some caregivers even say that they find the gestures they used to have with their children: “But it’s true that it reminded me of when my son was little. When we used to buy Pampers, but we put them on with pleasure” (FG7/C17).

#### 3.2.6. A Catalyst of Negative Elements

The term “urinary incontinence” was a real catalyst for negative feelings and words. Although caregivers and people in the general population did not associate the same keywords and feelings with the term “urinary incontinence,” they all relate to the topic very negatively. For caregivers, the subject is mostly associated with feelings: they cite embarrassment and shame. For the general population, UI is mostly associated with leakage, dependence, and loss of autonomy. Regarding associated feelings, caregivers were overwhelmingly divided between embarrassment, sadness, and anger. Those in the general population had regret and empathy as their primary feelings. Finally, caregivers often talked about the feelings their urinary incontinent loved one had. These were mostly shame, embarrassment, difficulty in accepting and living with the situation, and fear.

#### 3.2.7. A Complex Subject for Nursing Homes

Finally, according to the focus group participants, the management of UI is the responsibility and role of a nursing home. The majority of them have complete confidence in them regarding this subject: “I don’t know, I let them manage it” (FG7/C18); “it’s part of the establishment’s responsibility” (FG1/GP3). On the other hand, many participants have negative representations of what is done in these nursing homes: systematic use of protection, lack of mobility of residents and difficulties for the elderly to dare to say what they feel.

### 3.3. Social Representations of the Means of Protection

#### 3.3.1. A Little-Known Subject That Is the Subject of Debate

Like the subject of urinary incontinence, the subject of protective devices is not well known to the focus group participants. Indeed, despite some limited knowledge about the fact that there are different models, and the possibility of adapting the choice according to the needs of the urinary incontinent person, the vast majority of participants’ knowledge is based on what they see on television. In four focus groups out of seven, commercials were mentioned: “What you see in the commercial” (FG3/C6). The discussions also focused on the different solutions that could exist. The aim here was to understand whether the participants knew of solutions other than protection. In five focus groups out of seven, the participants talked about rehabilitation (mentioned 13 times). However, it was always the caregivers or the youngest members of the general population: “things to prevent you from getting to that point if you do it early enough, like strengthening the perineum, things like that” (FG2/GP5). Prevention was mentioned five times and operations three times. It is also important to note that in five focus groups out of seven, participants mentioned false beliefs, including the following: that there is only one solution to urinary incontinence, and that there is very little choice of pads (“There aren’t many brands either… there isn’t a wide range of choices…”-FG7/PG21; “There are not 36!”-FG3/C7; “Everything has to be done about it because we don’t have much choice”-FG2/GP5). Questions also came up several times in the exchanges, whether from caregivers or the general population: Are there different means of protection? Are there many brands? The means of protection are also a subject of debate among the participants: while the caregivers recognize a real evolution (“they are more and more sophisticated, and then quite… more beautiful anyway. And more practical” (FG4/C8), people from the general population have a very negative representation of protection: for some of them, wearing protection means “regressing” (FG5/GP15) and find it “degrading” (FG5/GP17).

#### 3.3.2. A Source of Inconvenience

During the focus groups, the participants underlined numerous problems related to protection, which, according to them, are a real source of inconvenience. Among the most cited inconveniences, we find the image given to others, the “overflowing”, the size problems, or the alterations of the skin condition, but also the alteration of the self-image: “She who had always been flirtatious, and who had small lace panties… it’s a blow to the femininity too” (FG7/C18).

#### 3.3.3. A Way to Increase Residents’ Empowerment

Finally, the means of protection are also represented as being able to increase the empowerment of nursing homes residents. For the majority of participants in the focus groups, and in five out of seven focus groups, it seems important that the urinary incontinent person be able to choose for him/herself what he/she wishes to have as protection, in the case obviously where he/she is not prevented from doing so by cognitive problems, that he/she be involved in the choice of the means of protection that is best adapted to him/her: “It is the person who must choose! It is the person who will feel and give his opinion” (FG2/GP5) or “The person himself, he can ask for advice but for me, it is up to them to choose, when they are able to choose” (FG3/GP9). However, in three focus groups out of seven, the caregivers consider that it is the caregivers who systematically choose the protections for their loved one: “I don’t think we asked her, as a mother, do you want this or that or there, I don’t think so” (FG1/C2). Moreover, for the vast majority of focus group participants (cited 15 times), the choice of protection method would be based on financial cost: “I would tend to go to the pharmacy first and then see if I could find something that is a bit… equivalent… there. To reduce the cost” (FG4/GP13). Only after cost came the criteria of comfort, absorption, and aesthetics.

### 3.4. Urinary Incontinence: A Source of Innovative Ideas

#### 3.4.1. Prevention and Education

First of all, it is primary prevention that is highlighted by the focus group participants. Indeed, in three of them, the participants raised the idea of focusing on prevention actions proposed from a very young age: “Why don’t we have certain sessions at school, when we play sports or whatever, that are more or less targeted, to work these muscles” (FG1/GP2). Education was then discussed in three of the seven focus groups. According to the caregivers and the general population, there is a real need for learning on this subject. It would be necessary to talk about this health problem earlier and to offer education to the youngest and to their parents: “all the courses related to health education to de-demonize it… well, to make it less taboo. Because I think that if we start talking about it to the youngest, it will be 50% of the work that has already been done” (FG3/GP10).

#### 3.4.2. Organizational Innovations

In five out of seven focus groups, participants gave ideas for organizational innovations to improve the management of UI and its consequences. For several of them, it would be interesting to set up personalized care, with, for example, the construction of a list of indicators that would be checked off during an individual interview, and which would allow the choice of the protection adapted to each resident: “pathways for each resident… where there would be, I don’t know, interviews, among all the solutions, which is the most adapted…in fact, there are plenty of indicators that could be constructed and afterwards try to point towards the best solution in the list that may exist…” (FG2/GP6). For others, it would be a question of organizing a systematic accompaniment to the toilets or even preserving walking: “preserving walking, that contributes to reducing the number of people with disabilities” (FG7/GP21).

#### 3.4.3. Technical Innovations

In four focus groups out of seven, technical and technological innovations were proposed by the participants, clearly linked to the means of protection. Among them, several of them proposed the invention of a thinner model of protection that could absorb urine. This innovative idea was proposed by women who use menstrual panties, who suggested having this same device for incontinence products: “As for girls, there are menstrual panties, I say to myself bah that must exist. We say to ourselves if there is a fabric that can stop blood, why can’t it stop urine, and therefore also odors” (FG1/C2). Others suggested improving the materials of incontinence products, with a view to reducing discomfort: “thinner and thinner textiles that protect more, but that’s… for less discomfort” (FG2/C4).

## 4. Discussion

As with any qualitative study, this research has a limit to its generalizability. Despite the precautions taken by the researchers, difficulties were encountered during the focus groups. For example, some participants sometimes dominated the exchanges, which could have led to off-topic discussions and a reluctance on the part of other participants, with shy or conformist profiles, to express their personal ideas [18]. Moreover, it seems important to note that, during a focus group, non-verbal expressions (gestures, hesitations, mimics, reactions…) play a primordial role in the exchanges and interactions between the participants [18]. These very valuable indications could not all be detected, because of the wearing of masks related to the COVID-19 pandemic. In order to obtain a large amount of non-verbal information, the researchers were trained to carefully observe the participants’ expressions. With the help of the facial micro expression grid proposed by Eckman [27], the participants’ attitudes and reactions could be evaluated. In addition, some biases can also be reported. For example, the physical presence of the researchers may have resulted in a moderator bias that unintentionally influenced the attitudes of the caregivers and the general population. This is known as the Hawthorne effect [28]. The training of the interviewers, and more specifically of the moderator, made it possible to limit this bias as much as possible.

In our qualitative focus group study, caregivers and people in the general population see UI as a disruptive element of daily life, on several levels: physical but also psychological. The literature describes UI as a problem that can lead to significant physical consequences for older people with urinary incontinence, such as altered skin condition, difficulty moving around, and falls [29]. A particular point was emphasized in the focus groups concerning the alteration of the self-image of elderly people suffering from urinary incontinence. This impairment is also found in a study published in 2019. In this research, 218 women with UI were asked about their body image using the “Body Cathexis” scale [30]. The results of this study showed that a majority of them had a negative body image of themselves. However, it seems important to point out that this study was not done with institutionalized elderly people. In 2014, a study looking at urinary incontinence and self-image showed that supporting older people on what UI represents for them [31] (shame, embarrassment, decreased self-esteem…) is as important as physical support (treatment, rehabilitation…).

This study also showed that UI could affect family relationships. During the focus groups, a link was made between urinary incontinence and intra-family conflicts. Two types of intra-family conflicts were highlighted: conflicts between the child and his or her urinary incontinent parent and conflicts between members of the same sibling regarding the management of their parent’s urinary incontinence in a nursing home. In the first case, several caregivers indicated that they sometimes had conflicts with their parent regarding their attitude. Indeed, the caregivers said they were irritated by their urinary incontinent mother’s behaviour. In the second case, it was conflicts between siblings concerning the management of their parent’s urinary incontinence in the nursing homes. This link has never been raised in the literature and it seems interesting to highlight it.

The study also showed the fact that UI was perceived by caregivers and the general population as a real transition in the lives of the people concerned. Indeed, it would be linked to the entry into old age. For them, it would be the beginning of a new and difficult stage. The parallel with the work of Meleis in 2000, and her theory of transition, leads us to emphasize this element. According to the theorist, “changes in people’s health and illness create a transition process [and they] tend to be more vulnerable to risks that may in turn affect their health” [32]. Yet the link between urinary incontinence in the elderly and transition has not been studied in the literature.

Like the topic of UI, the topic of protective devices appears to be unknown to caregivers and the general population. The focus group participants’ knowledge was limited and came mostly from what they had seen on television. This is the most common source of information available to the general population and is not very informative. A 2019 mixed-methods study by Smith aimed to determine the sources through which UI women sought and received information about absorbent products, and whether these sources were useful. His research showed that television was one of the primary sources of information about protection [33]. However, he also found that this information was not tailored to the needs of the individuals involved, who then made mistakes in their choice of protection.

The study also showed that for caregivers, as for the general population, protective devices were a source of inconvenience. In addition to all the physical consequences mentioned by the focus group participants, their self-image also seemed to be strongly altered. This link between wearing protection and alteration of self-image has also been made in the literature [30]. It would appear that body image is more negative, and that self-esteem and quality of life are lower in women using protection. Finally, the cost of the methods of protection was much mentioned by the participants. It was mentioned as a criterion for making a choice, but also as a problem. However, other criteria were also raised by caregivers and the general population: comfort, absorption, composition, and aesthetics. A few years ago, Getliffe studied the characteristics considered most important for a means of protection [34]. Here again, comfort, absorption and discretion were emphasized.

However, one point that seems essential to us has not been studied in the literature: it is the empowerment of urinary incontinent people. It seems important to caregivers and the general population that this person be able to choose his or her own means of protection, to the extent that he or she is able to do so. They believe that resident involvement in choosing the most appropriate protection is essential. In the literature, empowerment is defined as “a social process of recognizing, promoting and increasing people’s ability to meet their own needs, solve their own problems and mobilize the necessary resources” [35]. It would therefore be interesting, in these nursing homes, to lead the resident towards more decision-making and power to act.

In practice, social representations highlighted by this study show us that there is a real lack of knowledge on the subject, both on the part of caregivers and the general population. On the other hand, it seems possible to envisage possible actions to be put in place for a better understanding of this health problem. With more communication and information, the care and quality of life of the people concerned could be improved.

## 5. Conclusions

UI and means of protection are therefore the subject of many representations by caregivers and the general population. Although the subject is still very taboo in our society and a source of shame for the people concerned, it would seem possible to envisage primary and secondary prevention actions on a large scale. These could help to de-demonize the subject of urinary incontinence and limit the number of people affected by this health problem. Similarly, focus group participants described UI as a means of empowering residents. By choosing their means of protection, institutionalized elderly people could be active in the management of their UI. This could also reduce the very negative psychological impact of this disorder. Based on the results of this study and its perspectives, we can hope to develop strategies to improve the quality of life and dignity of institutionalized elderly people suffering from urinary incontinence, and to prevent the complications of this phenomenon so that future generations can avoid or live better with this health problem. This study provided a deeper understanding of the social representations of caregivers and people from the general population concerning urinary incontinence in institutionalized elderly people. It is part of a larger research program that also focuses on the representations of institutionalized elderly people suffering from this phenomenon and their carers.

## Figures and Tables

**Table 1:** Sociodemographic characteristics of the focus groups members.

	FG 1	FG 2	FG 3	FG 4	FG 5	FG 6	FG 7	TOTAL
**N**	7	6	5	7	5	6	5	41
**Age (Mean)**	50	50.5	50.2	63.6	52.8	56.2	59.8	54.73
**Women**	6	5	4	7	4	6	5	37
**Men**	1	1	1	0	1	0	0	4
**Caregivers**	3	2	2	4	2	3	3	19
**General Population**	4	4	3	3	3	3	2	22

## Data Availability

All data generated or analyzed during this study are available upon request from the corresponding author.
